# Emotional stimulation processing characteristics in depression: Meta-analysis of eye tracking findings

**DOI:** 10.3389/fpsyg.2022.1089654

**Published:** 2023-01-13

**Authors:** Genying Huang, Yafang Li, Huizhong Zhu, Hong Feng, Xunbing Shen, Zhencai Chen

**Affiliations:** ^1^College of Humanities, Jiangxi University of Chinese Medicine, Nanchang, China; ^2^Key Laboratory of Psychology of TCM and Brain Science, Jiangxi Administration of Traditional Chinese Medicine, Jiangxi University of Chinese Medicine, Nanchang, China; ^3^College of Acupuncture and Massage, Jiangxi University of Chinese Medicine, Nanchang, China

**Keywords:** depression, eye tracking, emotional stimuli, meta-analysis, attention bias

## Abstract

**Objective:**

To systematically evaluate the attentional bias in patients with depression toward emotional stimuli and to explore eye movement indicators and potential regulatory variables that can distinguish such patients from healthy individuals.

**Methods:**

Case–control studies regarding eye-tracking in major depressive disorder published in PubMed, Web of Science, ScienceDirect, The Cochrane Library, EBSCOhost, Embase, China National Knowledge Infrastructure, Wanfang, and VIP databases from database initiation until March 12, 2022 were included in the present meta-analysis. Two researchers independently screened the literature and performed data extraction. The quality of the literature was assessed using the Newcastle–Ottawa quality assessment scale.RevMan 5.4 software was used for Meta-analysis.

**Results:**

Overall, 14 studies were included, including 1,167 participants (*N*_depression_ = 474; *N*_healthy_ = 693). We found that (1) fixation duration was significantly lower for positive emotional stimuli in the depression group than that in the healthy group; however, for negative stimuli, the fixation duration was significantly more in the depression group than in the healthy group. No significant difference was observed in terms of neutral emotional stimuli between groups. (2) Patients with depression exhibited a significantly lower fixation count for positive emotional stimuli than healthy individuals, whereas the fixation count for negative emotional stimuli was significantly higher in the depression group than in the healthy group. No significant difference was found for neutral emotional stimuli between groups. (3) No significant difference was detected in terms of the first fixation duration of the positive, negative, and neutral emotional stimuli between groups. (4) subgroup analysis indicated that age effected fixation duration for positive emotional stimuli. In addition, age and the type of negative emotional picture (sad, dysphoric, threat, anger) effected fixation duration for negative emotional stimuli.

**Conclusion:**

Our research supports that patients with depression exhibit a negative attention bias toward emotional stimuli, and the fixation duration and fixation counts may be used as auxiliary objective indicators for depression screening.

## 1. Introduction

Depression is a common mental disorder with high recurrence and disability rates. The recurrence rate is 40–60% after the first episode, increasing to 90% after three episodes (van Rijsbergen et al., 2015). Depression affects >300 million people globally, constituting a major contributor to the global burden of disease (Beurel et al., 2020). Presently, medical practitioners utilize subjective diagnostic criteria to diagnose depression and evaluate patient symptoms during a clinical interview. Hence, the reliable diagnosis of major depressive disorder (MDD) using objective diagnostic criteria is challenging (Mitchell et al., 2009; Ding et al., 2019).

Attentional biases involved in the cognitive processing of emotional information may not only be a key factor in the etiology, maintenance, and recurrence of depression but also be vital for its diagnosis and treatment (Gotlib and Joormann, 2010; Disner et al., 2017). Eye tracking has proved to be an important technology for studying attentional bias and improving diagnostic techniques. A major advantage of eye tracking is that it directly and continuously records the allocation of attention over an extended period while multiple stimuli compete for attention (Figueiredo et al., 2019).

However, ambiguities remain in the present understanding regarding attentional bias in patients with depression. According to Beck's cognitive model of depression, biased acquisition and processing of negative information plays a primary role in the development and maintenance of depression (Beck, 2008; Disner et al., 2011). The model of emotion context insensitivity of depression proposes that patients with depression process emotional stimuli more slowly than healthy individuals and are consequently insensitive to both positive and negative emotional stimuli but more sensitive to neutral emotional stimuli (Rottenberg et al., 2005; Isaacowitz et al., 2006; Bylsma et al., 2008). As attentional bias in patients with depression is inconsistent, Suslow et al. performed a systematic narrative and meta-analytic review of eye tracking studies involving patients with clinical depression and examined specific aspects of attention or performance in specific tasks (Suslow et al., 2020). However, a limitation of this review was that only studies that used free viewing of images (*n* = 4) were included. Researchers utilizing eye tracking technology have typically used free-viewing tasks wherein participants are presented with multiple emotional stimuli in the form of emotional scenes, faces, and words or a combination of stimuli with the provision of limited instructions (van Rijsbergen et al., 2015). This provides data regarding the natural direct and continuous allocation of attention of the individuals in the free-viewing tasks (Hermans et al., 1999). Herein, we sought to aggregate and evaluate the available eye tracking research in the literature that used free-viewing studies based on images to better characterize clinical depression with regard to attentional biases for emotional stimuli and the potential moderators of their effects.

There are some studies of depression were not conducted under the assistance of psychiatrists (such as in the context of school counseling). Nevertheless, these studies do have some clinical meanings, and this kind of studies also was often seen in depression screening by using instruments of PHQ-9 and BDI as included criteria for clinical depression. Thus, these studies were included in the current meta-analysis. Furthermore, a subgroup analysis was performed to compare the population of depression based on ICD-10 and DSM-5 diagnostic criteria with the population diagnosed by the scales of PHQ-9 and BDI.

## 2. Methods

### 2.1. Search strategy

The key search terms used were “depression,” “depressive disorder,” “MDD,” “eye tracking,” “eye movement,” and “attention bias (AB).” A list of synonyms was created for each key term. The key terms were searched along with their synonyms in each separate database. The databases searched included PubMed, Web of Science, ScienceDirect, EBSCOhost, The Cochrane Library, Embase, China National Knowledge Infrastructure, Wanfang Data, and VIP database. The search window extended from when the databases were created to March 2022.

Two of the authors (Huang and Zhu) conducted independent systematic literature and manual searches of the reference list of the previous meta-analysis, and the retrieved articles were assessed.

### 2.2. Eligibility criteria

The inclusion criteria of this meta-analysis were as follows: (a) studies that published case–control analysis on eye tracking of individuals with depression, (b) patients aged ≥ 18 years who met the current international diagnostic criteria (Diagnostic and Statistical Manual of Mental Disorder 5 [DSM-5] or International Classification of Diseases) or were clinically diagnosed with depression as the case group and healthy individuals as the control group, (c) studies that used free-viewing tasks, and (d) studies that contained statistics and sufficient data for analyses.

Exclusion criteria were (a) review articles or meta-analyses, case reports, (b) eye movement studies examining patients with other disorders, and (c) articles that did not meet the diagnostic criteria for patients with depression in the DSM, ICD, or depression Diagnostic Scale, questionnaire (i.e., BDI, HAMD, PHQ-9).

### 2.3. Study selection

After removing duplicates, the titles and abstracts of the screened studies were independently screened by authors (Huang and Zhu) and any non-pertinent studies were excluded. The final list comprised studies accepted by both the authors. If the inclusion criteria were met, the full-text article was retrieved and screened to evaluate the available data for analysis.

### 2.4. Data collection process

Data extraction was independently performed by two authors (Huang and Zhu). Disagreements were resolved *via* discussion with another author (Li) until consensus was reached.

### 2.5. Data items

Data extraction included the following: (a) basic information regarding the included literature: title, first author, and publication time; (b) basic characteristics of the patients: diagnostic criteria for depression, average age, number of samples in each group, and stimulation materials; (c) eye tracking parameters applied in the free-viewing tasks: fixation duration, dwell time (in ms or %), and number of fixation time (in frequency or %).

### 2.6. Synthesis of results

Review Manager version 5.4.1. was used for statistical analysis and generation of forest and funnel plots. The effect value was expressed *via* standardized mean difference (SMD) along with 95% CI; larger the absolute value of SMD, larger the effect size. Chi-square test was used for heterogeneity test, and I^2^ ≤ 50% and *P* >0.10 were considered low heterogeneity. According to the research theory and purpose, this study adopted the random effect model (Borenstein et al., 2009).

### 2.7. Subgroup analysis

Q statistic was used to determine if subgroup analysis was necessary, as significant heterogeneity suggested that there were factors accounting for between-study variance in the effect size. A priori moderator variables included patient characteristics (age) and experimental characteristics (stimulus duration and emotional stimulus type).

### 2.8. Publication bias

To evaluate for publication bias and the stability of the results, we conducted sensitivity analysis and constructed separate funnel plots to determine fixation duration, number of fixation, and first fixation duration (Jin et al., 2015). We assessed the risk of bias using NOS Literature Quality Evaluation Scale for all the included studies (Stang, 2010).

## 3. Results

### 3.1. Study selection and characteristics

**Figure 1:**
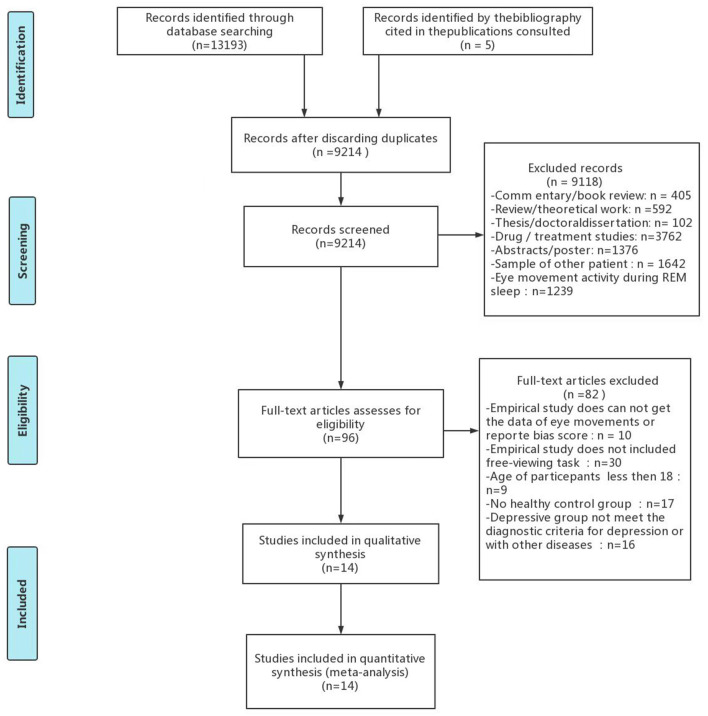
Flow diagram of the excluded and included studies.

The search strategy identified 13,198 articles, which was reduced to 9,214 after excluding duplicates. After screening the titles and abstracts of these articles, 96 were included for full-text screening and 82 were excluded. In total, 14 articles met the inclusion criteria and were included in the meta-analysis (see Figure 1).

These 14 eye tracking studies included 474 patients with depression and 693 healthy controls; participants were arranged in terms of age, and only two studies included older individuals. All the studies involved free-viewing tasks. In these studies, the participants were asked to simply observe emotional stimuli while they were shown faces or images. The emotional stimuli included being happy, positive, neutral, negative, sad, dysphoric, threatened, or angry. The duration of stimulus presentation ranged from 4 to 30 s. The outcome measures included early and later attentional processes (see Table 1).

**Table 1:** Studies included in the meta-analysis.

**References**	**Diagnose**	**N (MDD/HC)**	**Age (MDD/HC)**	**Type of stimuli**	**Affective qualities**	**Stimulus duration**	**Eye tracking outcomes**
Akram et al. (2021)	PhQ-9 ≥ 15	9/12	22.42 ± 7.44/25.16 ± 9.3	Scene		4 s	ac
Shuang et al. (2019)	DSM-5/HAMD ≥ 17	29/30	26.62 ± 7.12/24 ± 4.66	Face		10 s	a
Ding et al. (2019)	ICD-10	144/204	27.65 ± 9.50/27.46 ± 9.61	Scene		10 s	a
Eizenman et al. (2003)	SCID/BDI ≥ 16	8/9	36.9 ± 9.7/27 ± 5.7	Scene		10.5 s	ab
Isaac et al. (2014)	DSM-5	16/21	46 ± 8.6/44 ± 8.3	Face		10 s	ac
Kellough et al. (2008)	DSM-5/BDI-2/SCID	15/45	18–21/18–21	Scene		30 s	ab
Klawohn et al. (2020)	BDI-2 > 13/DSM-5/SCID	50/31	38.3 ± 12.1/34.9 ± 13.5	Face		6 s	a
Lanza et al. (2018)	ICD-10/HADS-D	15/20	74.6 ± 9.5/75.9 ± 7.5	Scene		10 s	ac
Li et al. (2016)	DSM-5/HAMD > 8	60/60	25.4 ± 7.2/24.2 ± 6.1	Scene		10 s	ab
Newman et al. (2019)	BDI-2/PHQ-9	14/28	29.5 ± 12.1/22.7 ± 10.6	Face		8 s	a
Noiret et al. (2015)	MADRS/BDI-2	20/62	71 ± 9.12/67 ± 5	Face		15 s	ab
Wang et al. (2022)	DSM-5/SCID/HAMD	48/104	30.67 ± 11/25 ± 5.09	Scene		8 s	ab
Wells et al. (2014)	DSM-5/BDI-2/SCID	26/47	31.3 ± 8.7/33.6 ± 11.2	Scene		30 s	ab
Zhang et al. (2020)	DSM-5/HAMD	20/20	30.5 ± 9.9/28.8 ± 4.4	Scene		4 s	abc

➀positive; ➁happy; ➂neutral; ➃negative; ➄sad; ➅dysphoric; ➆threat; ➇anger.

a, fixation duration; b, number of fixation time; c, first fixation duration.

HC, healthy control.

### 3.2. Publication bias

**Figure 2:**
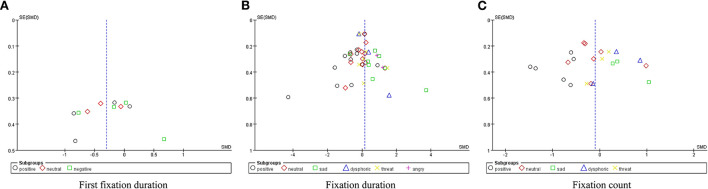
Funnel plots of publication bias of first fixation duration **(A)**, fixation duration **(B)**, and fixation count **(C)**.

**Figure 3:**
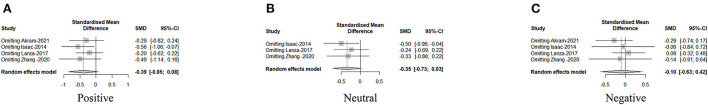
Sensitivity analysis of first fixation time of positive emotional stimulus **(A)**, neural emotional stimulus **(B)**, and negative emotional stimulus **(C)**.

**Figure 4:**
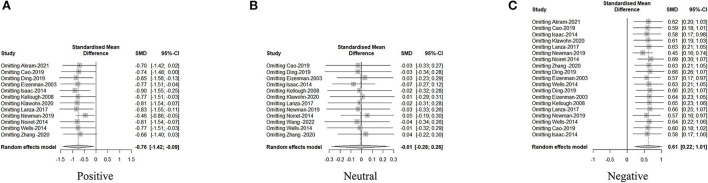
Sensitivity analysis of fixation duration of positive emotional stimulus **(A)**, neural emotional stimulus **(B)**, and negative emotional stimulus **(C)**.

**Figure 5:**
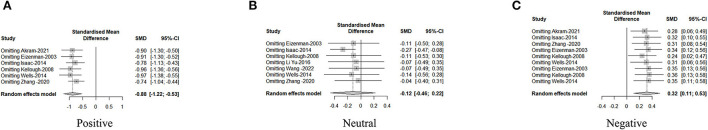
Sensitivity analysis of fixation count of positive emotional stimulus **(A)**, neural emotional stimulus **(B)**, and negative emotional stimulus **(C)**.

Publication bias was measured using funnel plots, and potential heterogeneity was eliminated using the one-by-one exclusion method. Publication bias minimally affected the results, and sensitivity analysis established the conclusion as relatively robust (see Figures 2–5).

### 3.3. Quality of individual studies

The quality of the included studies was determined using the 10-point Newcastle–Ottawa scale (NOS; Table 2). Of the analyzed studies, two scored 5 points, six scored 6 points, and six scored 7 points. Thus, the high quality of the literature indicates that publication bias exhibited minimal effect on the results.

**Table 2:** Quality of individual studies.

**References**	**Selection**				**Comparability**	**Exposure**		
	**Is the case definition adequate?**	**Representative of cases**	**Selection of controls**	**Definition of controls**	**Comparability of cases and controls based on the design or analysis**	**Ascertainment of exposure**	**Same method of ascertainment for cases and controls**	**Non-response rate**
Akram et al. (2021)	⋆		⋆		⋆	⋆	⋆	
American Psychiatric Association (2013)	⋆	⋆	⋆	⋆	⋆	⋆	⋆	
Ding et al. (2019)	⋆	⋆	⋆	⋆	⋆	⋆	⋆	
Eizenman et al. (2003)	⋆		⋆	⋆	⋆	⋆	⋆	
Isaac et al. (2014)	⋆	⋆			⋆	⋆	⋆	
Kellough et al. (2008)	⋆		⋆	⋆	⋆	⋆	⋆	
Klawohn et al. (2020)	⋆		⋆	⋆	⋆	⋆	⋆	
Lanza et al. (2018)	⋆		⋆	⋆	⋆	⋆	⋆	
Li et al. (2016)	⋆	⋆	⋆	⋆	⋆	⋆	⋆	
Newman et al. (2019)	⋆		⋆	⋆	⋆	⋆	⋆	
Noiret et al. (2015)	⋆	⋆	⋆	⋆	⋆	⋆	⋆	
Wang et al. (2022)	⋆	⋆		⋆	⋆	⋆	⋆	
Wells et al. (2014)	⋆	⋆	⋆	⋆	⋆	⋆	⋆	
Zhang et al. (2020)	⋆	⋆	⋆	⋆	⋆	⋆	⋆	

Of the analyzed studies, two scored 5 points, six scored 6 points, and six scored 7 points.

Thus, the high quality of the literature indicates that publication bias exhibited minimal effect on the results.

### 3.4. Synthesis of results

#### 3.4.1. Attention maintenance on emotional stimuli

The fixation duration for positive emotional stimuli was significantly lower in patients with depression than in healthy individuals (SMD = −0.71, *P* = 0.004), although for negative emotional stimuli, the fixation duration was significantly higher in patients with depression than in healthy individuals (SMD = 0.59, *P* = 0.0004) (Table 3). There was no significant difference for neutral emotional stimuli between groups (SMD = 0.01, *P* = 0.94). With regard to the number of fixations, patients with depression exhibited a significantly lower fixation count for positive emotional stimuli than healthy individuals (SMD = −0.87, *P* = 0.004), whereas the fixation count for negative emotional stimuli was significantly higher in the depression group than in the healthy controls (SMD = 0.32, *P* = 0.006). There was no significant difference for neutral emotional stimuli between groups (SMD = −0.13, *P* = 0.44). For the first fixation duration, no significant difference was observed for positive, negative, and neutral emotional stimuli between groups.

**Table 3:** Eye movement characteristics of patients with depression.

					**Heterogeneity**		
**Comparison**	**K**	***N***	**SMD**	**95% CI**	**df**	***p***	**I^2^**
**First fixation duration**							
Positive	4	133	−0.39	−0.85, 0.08	3	0.16	42%
Neutral	3	112	0.35	−0.73, 0.03	2	0.5	0%
Negative	4	133	−0.1	−0.63, 0.42	3	0.09	54%
**Fixation duration**							
Positive	12	895	−0.71^**^	−1.2, −0.23	11	< 0.00001	89%
Neutral	13	1,463	0.02	−0.20, 0.24	12	0.001	63%
Negative	19	1,506	0.59^***^	0.27, 0.91	18	< 0.00001	87%
**Fixation count**							
Positive	6	248	−0.87^***^	−1.21, −0.54	5	0.22	29%
Neutral	8	936	−0.18	−0.45, 0.09	7	0.02	58%
Negative	9	398	0.32^**^	0.09, 0.54	8	0.36	10%

^*^p < 0.05.

^**^p < 0.01.

^***^p < 0.001.

K, number of comparisons used in the analysis; N, number of participants; SMD, combined effect size; CI, confidence intervals; P, significance of between-study dispersion; I^2^, magnitude of between study dispersion.

According to Cohen's definition of effect size (where 0.2–0.5, 0.5–0.8, and >0.8 represent small, medium, and large effect sizes, respectively), the number of fixations for positive emotional stimuli had a large effect size, the fixation time for both positive and negative emotional stimuli had a medium effect size, and the total gaze number for negative emotional stimuli had a small effect size. This shows that the processing of emotional stimuli varies in patients with depression from that in healthy individuals and that the fixation time and number of fixation is important for processing positive and negative stimuli, and thus useful for diagnosing depression.

#### 3.4.2. Analysis of subgroups

According to the Cochrane evaluation system, ≤ 50% of heterogeneity in I^2^ is acceptable. Herein, the I^2^ of patients with depression in terms of fixation time for positive and negative emotional stimuli was 89% and 87% (*P* < 0.00001), respectively, indicating that regulatory variables may influence the effect size and that further analysis of the role of regulatory variables is warranted. Age, stimulus presentation time, emotional valence, and emotional stimulus type (emotional face and scene) potentially influences the fixation time of the participants. Therefore, we analyzed whether these four variables modulated fixation time (Tables 4, 5).

**Table 4:** Moderate effects of fixation duration on negative emotional stimuli.

					**Heterogeneity**		
**Subgroups**	**K**	**N**	**SMD**	**95% CI**	**df**	***p***	**I** ^2^
**Emotional valence**							
Sadness	8	397	0.86*	0.17, 1.56	3	0.07	57%
Dysphoria	3	438	0.4	−0.33, 1.14			
Threatening	6	575	0.21	−0.21, 0.64			
Anger	2	96	0.98^***^	0.55, 1.41			
**Age (years)**							
Young (< 60)	6	280	1.21^***^	0.51, 1.91	1	0.04	77.5%
Old (≥60)	2	117	−0.19	−1.28, 0.91			
**Presentation time**							
≤ 6 s	3	142	0.6^***^	0.25, 0.94	1	0.44	0%
>6 s	5	255	1.09	−0.11, 2.28			
**Stimulus type**							
Face	5	301	1.15^*^	0.05, 2.24	1	0.22	34.40%
Scene	3	96	0.41	0.00, 0.82			
**Diagnosis**							
Clinical diagnose	9	750	0.64^**^	0.23, 1.05	1	0.68	0%
Questionnaires	3	145	1.17	−1.28, 3.62			

^*^p < 0.05.

^**^p < 0.01.

^***^p < 0.001.

p, subgroup differences; K, number of comparisons used in the analysis; N, number of participants; SMD, combined effect size; CI, confidence intervals; P, significance of between-study dispersion; I^2^, magnitude of between study dispersion.

**Table 5:** Moderate effect of fixation duration under positive emotional stimulation.

					**Heterogeneity**		
**Subgroups**	**K**	**N**	**SMD**	**95% CI**	**df**	***p***	**I** ^2^
**Age**							
Young (< 60)	10	778	−0.85^**^	−1.44, −0.26	1	0.07	70.60%
Old (≥60)	2	117	−0.18	−0.58, 0.23			
**Presentation time**							
≤ 6s	3	142	−1.05^*^	−1.99, −0.10	1	0.44	0%
>6s	9	753	−0.61^*^	−1.19, −0.03			
**Stimulus type**							
Face	5	301	−0.89	−1.94, 0.17	1	0.67	0%
Scene	7	594	−0.63^*^	−1.17, −0.09			
**Diagnosis**							
Clinical diagnose	9	750	−0.40	−0.83, 0.04	1	0.18	45.6%
Questionnaires	3	145	−1.96	−4.17, 0.26			

^*^p < 0.05.

^**^p < 0.01.

^***^p < 0.001.

p, subgroup differences; K, number of comparisons used in the analysis; N, number of participants; SMD, combined effect size; CI, confidence intervals; P, significance of between-study dispersion; I^2^, magnitude of between study dispersion.

For fixation time in response to negative stimulus, subgroup analyses showed that (a) The effect size of the young and middle-aged group (SMD = 1.21) was larger than that of the older group (SMD = −0.19), with the effect size of the young and middle-aged group being significant (*P* = 0.0007). (b) The effect size (SMD) of the sadness and anger stimuli was 0.86 and 0.98, respectively, with that of the sadness stimuli being significant (*P* = 0.02) and that of anger stimuli being highly significant (*P* < 0.00001). The effect size of the threatening and dysphoric pictures was not significant. (c) There were no significant moderating effects on the time of stimulus presentation or the type of emotional stimuli.

For fixation time in response to positive stimulus, subgroup analyses showed that the effect size of the young and middle-aged group (SMD = −0.85) was larger than that of the older group (SMD = −0.18), with the effect size of the young and middle-aged group being significant (*P* = 0.005). The time of stimulus presentation and the type of emotional stimuli exhibited no significant interaction effect.

## 4. Discussion

The eye-tracking paradigm has gained more and more importance in depression research in recent years. Eye-tracking devices trace the time course of visual attention by almost continuously recording eye movements, thus allowing for revealing the characteristics of attention processing (Bianchi and Laurent, 2015; Lazarov et al., 2018; Wang et al., 2022). Fixation eye movement indicators are widely used in attention bias research of depression, mainly including first fixation time, fixation duration, fixation number, etc. The first fixation duration generally represents the characteristics of early processing, which can reflect the attention attraction of the emotional stimulus (Rantanen et al., 2021). The fixation duration generally represents the processing difficulty, a longer fixation duration indicates a higher level of attention and a corresponding greater processing difficulty (Isaac et al., 2014). The number of fixations primarily reflects the information extraction efficiency and information processing ability of the cognitive process (Roux et al., 2016; Akram et al., 2020). In this meta-analysis, published evidence regarding the characteristics of clinical depression with respect to attentional biases in response to emotional stimuli and their potential moderator effects was analyzed. The primary outcome of this study was that in patients with depression, the fixation time and fixation count for negative and positive emotional stimuli were considerably higher and lower than those in healthy patients, respectively. Age was an important regulatory variable of fixation time for positive emotional stimuli, and age and type of negative emotional stimuli were regulatory variables for the fixation time of negative emotional stimuli.

### 4.1. Patients with depression showed attentional maintenance of negative emotional stimuli

Fixation duration has been considered indicative of the depth of individual cognitive processing and maintenance of a stimulus. Our meta-analysis identified that the effect sizes of fixation time for negative and positive emotional stimuli reached 0.59 and −0.71, respectively. This suggests moderate differences between patients with depression and healthy individuals in terms of attention maintenance for negative and positive emotional stimuli. Our results are similar to those reported by Suslow et al. (2020). A study regarding the “antireward center” mechanism of depression reported that the abnormal increase in the activity of the lateral bridle nucleus neurons in patients with depression does not only prevent the processing of positive emotional information but also inhibits the positive processing of reward stimuli. Furthermore, the insensitivity of such patients toward reward stimuli impacts the goal-directed attentional system (Gold and Kadriu, 2019). This confirms that patients with depression exhibit insufficient bias toward positive emotional information and excessive cognitive processing and maintenance of negative emotional information.

The number of fixations primarily reflects the information extraction efficiency and information processing ability of the cognitive process. Patients with depression looked at fewer positive images compared with healthy individuals. The effect sizes of the fixation times for negative and positive emotional stimuli (SMD = 0.32 and −0.87, respectively) suggest moderate differences between patients with depression and healthy individuals with respect to attention maintenance in response to negative and positive emotions. The results of event-related potential studies (Ke et al., 2020) also confirmed that patients with depression exhibited electroencephalogram characteristics of prolonged response time when processing negative emotional information. This indicates that patients with depression extract negative emotional information with low efficiency and poor information processing ability.

The first fixation duration chiefly reflects the early attention given to directional processing characteristics (Grafton et al., 2016). No significant group differences between patients with depression and healthy individuals were observed for initial attention orientation to negative, neutral, or positive emotional stimuli, consistent with previous findings (Grafton et al., 2016). Thus, patients with depression appear to not be characterized by alterations during early orientation toward emotional stimuli (Suslow et al., 2020).

Difficulties in separating negative emotional information may be an important reason underlying the onset and maintenance of depression. In addition, functional brain imaging studies have demonstrated that the intensity of cortical activity, such as in the right prefrontal and right parietal lobe, weakens in depression, and that this cortical function is primarily associated with functions such as attentional selection and transfer (Skinner et al., 2018; Yaroslavsky et al., 2019). However, the electrophysiological indicators involved in the two-stage model framework of emotional attention regulation suggest that patients with depression mostly exhibit an enhanced P3 amplitude and negative bias during early attention allocation (Ao et al., 2020). However, controversy remains concerning the occurrence stage of negative attention bias, and this may be related to the lack of consistency in the emotional stimulus types and presentation time in the various studies. Hence, more evidence regarding the onset stage of negative attention bias is required.

### 4.2. Age and negative emotional type affect attention processing in patients with depression

The subgroup analysis revealed a significant effect of age. Patients with depression who were young and middle-aged observed more negative emotional stimuli (SMD = 1.21) and less positive emotional stimuli (SMD = −0.85) than healthy individuals, although no significant difference between older healthy individuals and older patients with depression was observed. According to the socioemotional selectivity theory (Carstensen et al., 1999; Carstensen, 2006), older adults prioritize emotion-regulatory goals. Furthermore, individual attentional sensitivity to negative stimuli gradually decreases with age (Mather and Carstensen, 2003; Lu et al., 2017); structural and functional changes in the brain also follow the same trajectory of behavioral change (Luna and Sweeney, 2004). These findings confirm the presence of emotional attentional biases in patients with MDD that are influenced by age. Attention bias modification (ABM) techniques have been confirmed to effectively improve cognitive bias modification procedures in depression (Vazquez et al., 2016). Consequently, clinicians should select the corresponding intervention based on patient age to improve clinical efficacy of ABM techniques. However, as there are presently relatively few empirical studies regarding eye movements in older patients with depression, more studies are warranted to confirm these preliminary results.

The subgroup analysis revealed a significant effect of negative emotional type. For instance, the effect size of fixation time on the emotional stimuli such as sadness and anger was 0.86 and 0.98, respectively, indicating a more significant attentional bias toward these stimuli. Daily negative emotions persist in depression, especially constant sadness (American Psychiatric Association, 2013). In addition, patients with MDD exhibited inflexibility in sadness and avoidance, which were associated with weaker FC between the right sgACC and pregenual/dorsal anterior cingulate (pg/dACC) (Schwartz et al., 2019). Furthermore, the emotions sadness and anger have been highly associated with depression (Koh et al., 2002; Ou and Hall, 2018). According to clinical surveys, the irritability rate of patients with depression can be as high as 30–85% (Bodner et al., 2018), and the DSM-5 views anger as a marker of depression in children and adolescents (American Psychiatric Association, 2013). Anger and irritability are prevalent in adults with depression and hinder the correction of depression cognitive bias more strongly than other negative emotions (Perlis et al., 2009). Our results are similar to those of previous studies, thus verifying that sadness and anger play an important etiological role in developing depression, which further helps simulate the treatment of emotion regulation and attentional bias correction in depression.

The interaction effect of the stimulus presentation time on emotional stimuli was not significant. Our results are similar to those of a meta-analysis conducted by Peckham who found no evidence regarding the significant moderation of biased attention toward negative information by stimulus presentation time in emotional Stroop or dot probe tasks (Peckham et al., 2013). Total gain duration and early stage of automatic attention allocation are both indicators of attention bias. Total gain duration usually used as attention maintenance index to evaluate negative attention bias (Duque and Vázquez, 2015; Lazarov et al., 2018), and it is reported that there was a significant main effect of stimulus presentation time on total gain duration (Wells et al., 2014). Previous ERP studies have demonstrated that automatic attention attraction by emotionally stimuli could reliably enhance the P1 amplitudes in the dot-probe task, which has been associated with rapid and unconscious bottom-up attention allocation (Luck and Kappenman, 2012), representing a facilitated perception in response to the emotional stimulus (Brosch et al., 2008; Liu et al., 2015). Eye tracking study in patients with depression have found a bias in the initial orientation to depression-related images (Sears et al., 2011). There is a lack of consensus on whether negative attentional bias occurs at early attention stages. It is demonstrated that patients with depression show a attentional bias toward congruent sadness at 100 ms, indicating that negative attention bias occurs in the early stage of attention (Trapp et al., 2018; Ao et al., 2020). While Cao found that the negative attention bias of patients with depression appeared after 6 s, suggesting that attention bias does not occur until the later stage of cognitive processing (Shuang et al., 2019). Therefore, we suggest that stimulus presentation time may be an influencing factor of emotional stimulus processing in patients with depression. But our study results are inconsistent with these results, probably owing to the stimulus material or material presentation time grouping. Therefore, these factors should be controlled and further discussed in the future.

In addition, attention bias mechanism has been suggested to mostly occur when processing social information stimuli. Faces showing emotions reflect the emotional state of others and thus contain more social information than emotional scene pictures and are more likely to attract the attention of patients with depression (Gilboa-Schechtman et al., 2004). However, there was no significant difference in the attentional bias toward emotional faces or scenes among the patients with depression included in this study. According to Leyman's research on attentional bias using forward-placed and inverted emotional faces, it is the emotional titer expressed by stimulus materials that causes individual attentional bias (Leyman et al., 2011) and when we looked back at the emotional scenes that were included in the study, we found that some of the emotional scene materials contained both characters and scenes, which is the same emotional valence as the emotional faces. Our results may support the idea that it is the emotional valence expressed by the stimulus material that causes attention bias in patients with depression. We suggest that future studies can compare the attention bias of depressed patients to emotional faces and emotional scenes to verify this view.

Eye movements are sensorimotor functions of the brain, and the effect of gender on attention patterns has been shown in autism spectrum disorders (Harrop et al., 2018). Classification of emotions based on EEG and eye movement signals also agree that emotion classification differs between male and female, and there are larger differences of brain activities between male and female for sad, which is closely associated with depression (Bao et al., 2019). Studies on attention bias in healthy adults also show that attention bias is influenced by gender and mainly manifests in the regulation function of negative stimulus avoidance (Pintzinger et al., 2016). Previous studies on attentional bias in depression have shown that sex differences in the subjects may lead to different initial attentional bias, and female patients with depression are more likely to have an initial attentional bias toward negative information (Sears et al., 2011; Li et al., 2016) and compared with men, women also have longer depressive episodes and are also presented higher levels of inflammatory, serotonergic markers, and neurotrophic (Oquendo et al., 2013; Labaka et al., 2018). Thus, it is important to consider gender as a biological variable in clinical depression studies, which may facilitate the development of more effective diagnostic methods and pharmacotherapies for depression.

Furthermore, the results of subgroup analysis of diagnostic methods showed that there was no significant difference in the negative attention bias of depressive patients diagnosed based on clinical diagnostic criteria and questionnaire, and we preliminarily believed that the diagnostic methods did not have much influence on the results. However, in order to ensure the reliability and extensibility of the research results, the inclusion criteria should be more strictly established in future studies.

### 4.3. Limitations

Several limitations must be considered before translating these results into clinical settings. First, we only analyzed the attention-processing characteristics of emotional stimuli in patients during the onset of depression. Thus, our study did not summarize these characteristics in the different stages of depression, such as first recurrence and disease duration, and did not comprehensively evaluate the value of this parameter for diagnosing depression. Second, only a limited number of studies was included in the meta-analysis for older patients with depression, thereby potentially reducing the reliability of its conclusions. To stimulate the clinical application of attention bias correction technology, future studies should observe and compare the attention-processing characteristics of patients with depression on emotional stimuli at different ages and stages and degrees of the disease, explore more indicators, such as saccade, and further evaluate the application value of eye movement indicators in diagnosing depression.

## Data availability statement

Publicly available datasets were analyzed in this study. This data can be found here: https://drive.google.com/file/d/1d1_w1sqk78UIRZLj8xBVDikt0w6_AtgS/view?usp=share_link.

## Author contributions

GH and YL convinced and designed this study. GH, YL, and HZ search and screen the literature. ZC and XS processed methodological analysis. GH and ZC processed data and drafted the manuscript. ZC and YL reviewed and modified the manuscript. All authors read and approved the final manuscript.
